# Candidate tumor suppressor B-cell translocation gene 3 impedes neoplastic progression by suppression of AKT

**DOI:** 10.1038/cddis.2014.550

**Published:** 2015-01-08

**Authors:** Y-C Cheng, P-H Chen, H-Y Chiang, C-S Suen, M-J Hwang, T-Y Lin, H-C Yang, W-C Lin, P-L Lai, S-Y Shieh

**Affiliations:** 1 Institute of Biomedical Sciences, Academia Sinica, 128 Section 2, Academia Road, Taipei 115, Taiwan; 2 Department of Pathology, National Taiwan University Hospital, No. 7, Chung Shan S. Road., Taipei 100, Taiwan

## Abstract

*BTG3* (B-cell translocation gene 3) is a p53 target that also binds and inhibits E2F1. Although it connects two major growth-regulatory pathways functionally and is downregulated in human cancers, whether and how BTG3 acts as a tumor suppressor remain largely uncharacterized. Here we present evidence that BTG3 binds and suppresses AKT, a kinase frequently deregulated in cancers. BTG3 ablation results in increased AKT activity that phosphorylates and inhibits glycogen synthase kinase 3*β*. Consequently, we also observed elevated *β*-catenin/T-cell factor activity, upregulation of mesenchymal markers, and enhanced cell migration. Consistent with these findings, BTG3 overexpression suppressed tumor growth in mouse xenografts, and was associated with diminished AKT phosphorylation and reduced *β*-catenin in tissue specimens. Significantly, a short BTG3-derived peptide was identified, which recapitulates these effects *in vitro* and in cells. Thus, our study provides mechanistic insights into a previously unreported AKT inhibitory pathway downstream of p53. The identification of an AKT inhibitory peptide also unveils a new avenue for cancer therapeutics development.

BTG3 is a member of the B-cell translocation gene/transducer of ErbB2 (BTG/Tob (transducer of ERBB2)) antiproliferative protein family that also includes BTG1, BTG2/PC3/Tis21 (TPA-induced sequence 21), BTG4, Tob1, and Tob2.^1^ The members of this protein family are characterized by a conserved N-terminal domain containing box A and box B signature motifs, and a variable C-terminal domain.^2^ Overexpression of BTG/Tob proteins is associated with inhibition of cell cycle progression, which is mostly mediated by their conserved N-terminal domain. For example, BTG2 inhibits G1-to-S progression via the downregulation of cyclin D1 and cyclin E,^3^ whereas BTG3 binds and inhibits E2F1, a transcription factor important for S-phase entry.^4^ Both BTG2 and BTG3 are transcriptional targets of the tumor suppressor p53, thus linking this family of proteins with stress response.^4, 5^ In addition, the N-terminal conserved domains of the BTG/Tob proteins are also known to interact with CAF1 (CCR4-associated factor 1), thereby modulating mRNA deadenylation^6, 7^ or cell proliferation.^8^ Less is known regarding the functions of the structurally diverse C terminus. The C termini of BTG1 and BTG2 interact with the protein arginine methyltransferase PRMT1 (protein arginine methyltransferase 1).^9^ More recently, the C terminus of BTG3 was found to bind CHK1 (checkpoint kinase 1) and safeguard genomic stability.^10^ Despite these findings, the biological relevance of this domain remains mostly uncharacterized. Downregulation of BTG3 was found in human cancers,^11, 12, 13, 14^ implicating a possible role as a tumor suppressor.

In contrast to the BTG/Tob proteins, the Ser/Thr kinase AKT acts as a prosurvival and proproliferation factor. AKT is involved in the regulation of many cellular processes via various downstream effectors such as mammalian target of the rapamycin (mTOR) in protein synthesis^15^ and the transcription factors nuclear factor- *κ*B and FOXO (forkhead box O) in cell survival.^16, 17^ Importantly, it allows the stabilization and subsequent nuclear localization of *β*-catenin by phosphorylating and inhibiting glycogen synthase kinase 3*β* (GSK3*β*), thus connecting *β*-catenin with cell growth and migration.^18, 19^ The activation of AKT requires the phosphorylation of its Thr308 and Ser473 residues by PDK1 (phosphoinositide-dependent kinase-1) and mTORC2 (mammalian target of rapamycin complex 2), respectively,^20, 21^ which is mediated by the binding of the N-terminal pleckstrin homology (PH) domain to membrane phosphatidylinositol-3,4,5-trisphosphate. Upstream stimuli such as growth factors activate receptor tyrosine kinase, which triggers the activation of phosphoinositide 3-kinase (PI3K) and leads to the generation of PIP3. The tumor suppressor PTEN (phosphatase and tensin homolog) converts PIP3 to phosphatidylinositol-4,5-bisphosphate, thus antagonizing PI3K-AKT signaling.^22^

The epithelial-to-mesenchymal transition (EMT) in tumors is characterized by a decrease in cell–cell adhesion and an increase in cell invasion and motility, and is believed to contribute to metastasis.^23, 24^ Many signaling events have been shown to be involved: among them, Wnt signaling inhibits GSK3*β*-mediated phosphorylation and degradation of *β*-catenin. The increased *β*-catenin translocates to the nucleus, where it induces the expression of genes encoding proteins such as vimentin and fibronectin that specify EMT.^25^

We demonstrated previously that BTG3 binds and inhibits E2F1, and therefore preventing S-phase entry and also maintaining G2/M arrest.^4^ We also showed that in normal human fibroblasts, BTG3 functions as a fail-safe anticancer barrier.^14^ Regardless of these findings, several issues remain unaddressed. For example, despite the enhanced E2F1 activity and its link with the induction of apoptosis, cell death was never observed in BTG3-depleted cells. Here we delineated further the effects of the loss of BTG3 and demonstrated its role in safeguarding the AKT-GSK3*β* pathway, and, by crosstalk with the Wnt/*β*-catenin pathway, its role as a barrier to tumor progression.

## Results

### BTG3 interacts with and inhibits AKT

**Figure 1:**
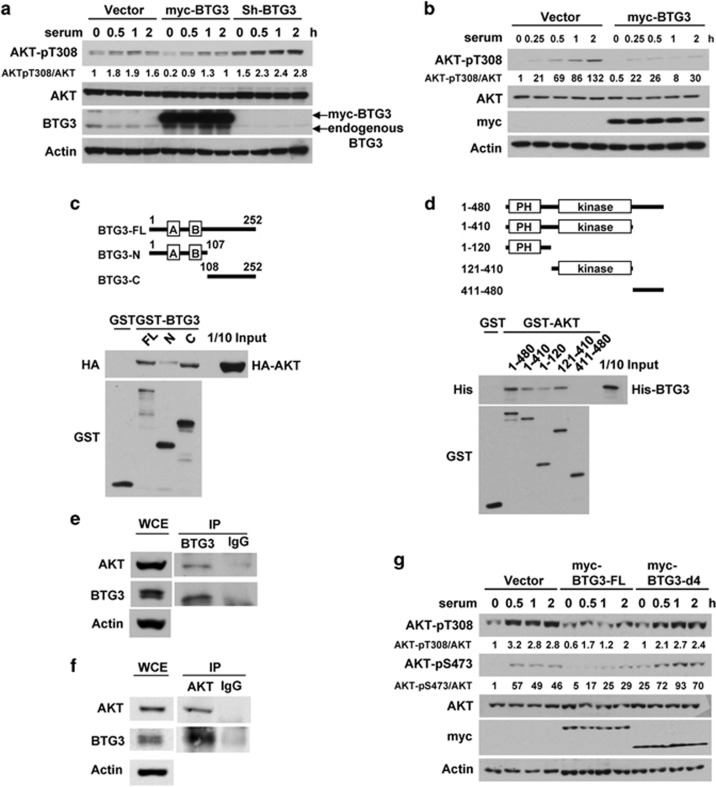
BTG3 binds to and inhibits AKT. (**a**) BTG3 overexpression inhibited AKT activation by serum, whereas its depletion elevated it. The 293 T cells were transiently transfected with a vector expressing myc-tagged BTG3 (myc-BTG3) or BTG3-targeting shRNA (shBTG3). Cells were serum starved for 24 h and collected at the indicated times after the addition of serum. Lysates were analyzed by western blotting using the indicated antibodies. (**b**) AKT activation was inhibited by ectopically expressed myc-BTG3 in DU145 prostate cancer cells. (**c**) The BTG3 C-terminal domain interacts with AKT. Recombinant GST or GST-fused full-length, C-terminally truncated or N-terminally truncated BTG3 was incubated with 293 T lysates expressing HA-tagged AKT. Proteins that were pulled down by the GSH beads were analyzed using western blotting. (**d**) The N-terminal and the core domains of AKT mediated the interaction with BTG3. GST pull-down assays were performed using purified recombinant His-BTG3 and GST or GST fused with full-length or truncated AKT. (**e** and **f**) Endogenous AKT and BTG3 interact. Co-immunoprecipitation of BTG3 and AKT from 293 T lysates was performed with either anti-BTG3 (**e**) or anti-AKT (**f**) antibody. (**g**) Overexpression of the full-length BTG3 but not the interaction-deficient d4 mutant inhibited the activation of AKT by serum in 293 T cells. BTG3-d4: amino acids 1–146. Image quantification was performed using a MetaMorph (Molecular Device, Sunnyvale, CA, USA)

The AKT signaling pathway controls cell proliferation, growth, and survival, and is frequently deregulated in cancers as a result of gene mutation, amplification, or loss or mutation of its negative regulator PTEN.^26^ As BTG3 has been implicated in tumor suppression,^14, 27^ we wondered whether it would be involved in the regulation of AKT. To determine whether BTG3 has an impact on AKT signaling, we first performed overexpression or knockdown experiments in 293 T cells, using AKT phosphorylation as an indicator. As shown in Figure 1a, overexpression of BTG3 suppressed AKT Thr308 phosphorylation (pT308), whereas its downregulation using a specific short hairpin (sh) RNA had the opposite effect. Similarly, overexpression of BTG3 in the prostate cancer cell line DU145 dampened serum-induced AKT activation (Figure 1b), suggesting that this is not a cell-type-specific effect. *In vitro*, the C-terminal domain (amino acids 108–252) of recombinant BTG3 interacted with AKT through the N-terminal and central regions that host the PH and kinase domains (Figures 1c and d). Further analysis of the BTG3 C terminus identified the region between amino acids 147 and 252 that mediates this interaction (Supplementary Figure S1). The interaction between endogenous BTG3 and AKT in 293 T cells was also detected by co-immunoprecipitation using the anti-BTG3 antibody (Figure 1e), verified by reciprocal immunoprecipitation using the anti-AKT antibody (Figure 1f). Although full-length BTG3 significantly inhibited AKT activation, the BTG3-d4 mutant (amino acids 147–252 deleted) that lacks the AKT interaction domain could not (Figure 1g and Supplementary Figure S1), suggesting that direct interaction is required for the inhibition.

### BTG3 impedes AKT recruitment to the plasma membrane

**Figure 2:**
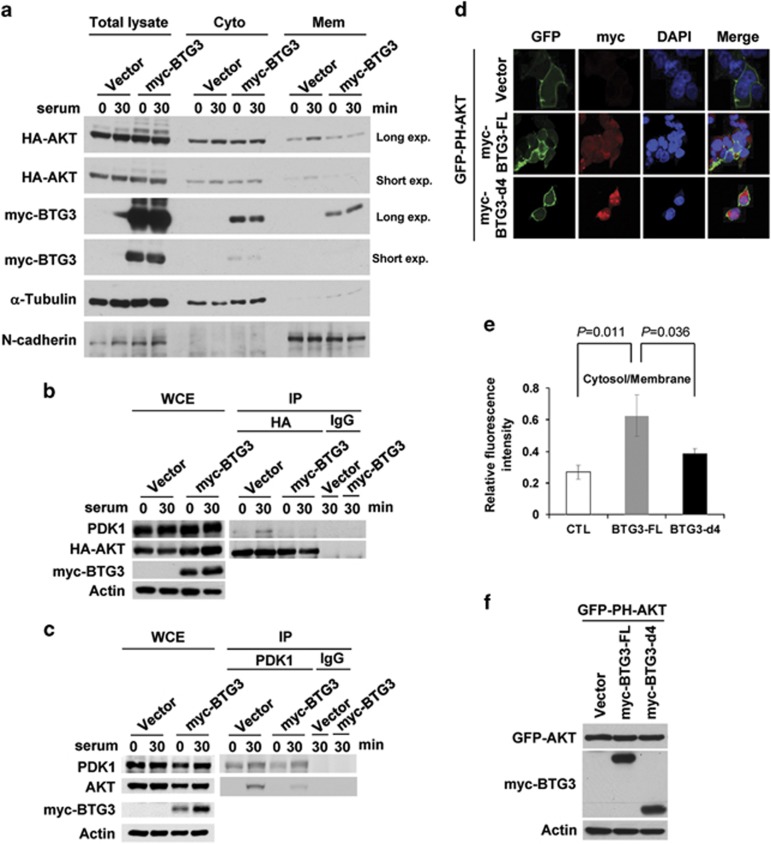
BTG3 inhibits the recruitment of AKT to the plasma membrane and its interaction with PDK1. (**a**) Ectopically expressed myc-BTG3 interfered with the membrane localization of AKT after serum stimulation. The 293 T cells were transfected with HA-AKT and myc-BTG3 and analyzed by immunoblotting after cell fractionation. (**b** and **c**) Overexpression of BTG3 blocked AKT-PDK1 interaction. The 293 T cells were transfected as in (**a**) and the presence of PDK1 in immunoprecipitated HA-AKT (**b**) or AKT in immunoprecipitated PDK1 (**c**) was determined by immunoblotting. (**d**) BTG3 impeded membrane localization of the AKT PH domain. Confocal microscopy was performed on 293 T cells transfected with a construct expressing GFP fused to the AKT PH domain, together with either full-length BTG3 (FL) or the BTG3-d4 mutant. The ratios of fluorescence intensity in the cytosol *versus* the cell membrane were determined and are shown in (**e**). (**f**) Relative expression levels of the two BTG3 proteins used in (**d**)

AKT is phosphorylated and activated at the plasma membrane by its upstream kinases. In an attempt to determine the underlying mechanism for BTG3-mediated inhibition of AKT, we examined the level of membrane-associated AKT in the presence or absence of ectopically expressed BTG3. Upon serum stimulation, AKT at the plasma membrane was increased in control vector-transfected 293 T cells. Such an increase was abolished in cells with ectopically expressed BTG3 (Figure 2a). Furthermore, serum stimulation enhanced the interaction of AKT with its upstream kinase PDK1, and such interaction was disrupted by ectopically expressed BTG3 (Figures 2b and c).

To provide further support for the idea that BTG3 mediates the suppression of AKT membrane localization, we then used a fusion construct that expressed green fluorescent protein (GFP) fused to the PH domain of AKT (GFP-PH-AKT).^28^ In 293 T cells, which express high PI3K activity, GFP-PH-AKT resided mostly in the plasma membrane (Figure 2d). Upon coexpression of the full-length BTG3, the membrane localization of GFP-PH-AKT was partially disrupted (Figures 2d and e). Such interference was not observed when the AKT-interaction-defective mutant d4 was used (Figures 2d and f), indicating that the suppression was most likely mediated through direct interaction. As a control, BTG3 did not significantly impact the membrane localization of the GFP-fused PH domain of Grp1 (general receptor of phosphoinositides 1) (GFP-PH-Grp1) (Supplementary Figure S2), suggesting that the regulation is specific to PH-AKT. Collectively, our data suggest that BTG3 inhibits the activation of AKT, at least in part, by preventing AKT from binding to the plasma membrane.

### BTG3 suppresses AKT-GSK3*β*-*β*-catenin signaling

**Figure 3:**
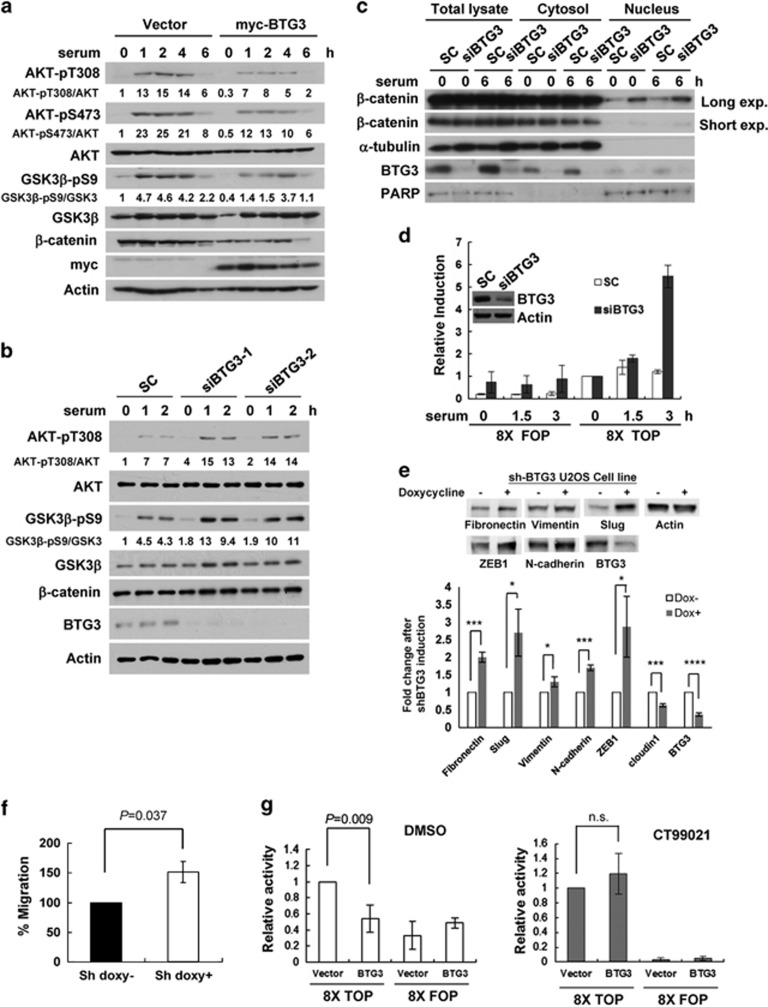
BTG3 suppresses EMT by antagonizing the AKT-GSK3*β*–*β*-catenin signaling axis. (**a** and **b**) Overexpression of BTG3 led to diminished GSK3*β* phosphorylation (**a**), whereas BTG3 depletion with siRNA enhanced it in U2OS osteosarcoma cells. (**b**) Cells were starved for 30 h and collected at the indicated times after serum stimulation. Lysates were analyzed by western blotting using the indicated antibodies. siBTG3-1 and -2 are siRNAs targeting two different sequences in BTG3. (**c**) Biochemical fractionation showing increased levels of *β*-catenin in the nuclear fractions of BTG3-downregulated U2OS cells. (**d**) The transcriptional activity of *β*-catenin/TCF was increased in BTG3-knockdown cells. U2OS cells were transfected with wild-type (8 × TOP) or mutant (8 × FOP) *β*-catenin/TCF reporters, serum starved for 24 h, and collected at the indicated times after the addition of serum. All luciferase activities were normalized to the co-transfected internal control and expressed as fold induction relative to the wild-type reporter in serum-starved cells (0 h). The mean±S.D. of three independent experiments is shown. (**e**) Increased expression of markers indicative of EMT in Tet-On shBTG3 U2OS cells after the induction of BTG3 shRNA expression by doxycycline. Immunoblot analysis was performed 72 h after the addition of doxycycline. Expression was quantified and mean±S.D. from three independent experiments is shown. *P<0.05, ***P<0.001, ****P<0.0001. (**f**) Enhanced migration of Tet-On shBTG3 U2OS cells after the induction of BTG3 shRNA expression by doxycycline (doxy). The mean±S.D. of three independent duplicated experiments is shown. (**g**) Inhibition of *β*-catenin/TCF activity by BTG3 requires GSK3*β* activity. The 293 T cells were transfected with the *β*-catenin/TCF reporters together with BTG3 or the control vector. Cells were treated with DMSO (solvent) or 2 *μ*M CT99021 (a GSK3-specific inhibitor) for 3 h before collection (*n*=3). NS, nonsignificant

To understand further the functional impact of BTG3-AKT interaction, events that lie downstream of AKT activation such as AKT-GSK3β signaling were investigated. Concordant with AKT inhibition (Figure 1b), overexpression of BTG3 in U2OS cells led to decreased GSK3*β* phosphorylation. This enhanced GSK3*β* activity culminated in reduced levels of *β*-catenin, likely as a result of enhanced degradation^29^ (Figure 3a). Conversely, after depletion of BTG3, the AKT and GSK3*β* phosphorylation levels were enhanced (Figure 3b) and the amount of nuclear *β*-catenin increased, as demonstrated by cell fractionation (Figure 3c). The increased nuclear presence of *β*-catenin after BTG3 depletion also markedly enhanced the activity of the *β*-catenin/T-cell factor (TCF) reporter (Figure 3d) as well as the production of its endogenous downstream targets, fibronectin, and vimentin (Figure 3e). Levels of Slug, ZEB1 (zinc-finger E-box-binding homeobox 1), and N-cadherin were also increased. As fibronectin, vimentin, and the other molecules are markers of EMT,^25^ cell migration was assessed using a transwell assay. A 50% increase in cell migration was observed after downregulation of BTG3 by inducible shRNA (Figure 3f). Of note, levels of E-cadherin were not significantly changed by BTG3 depletion in U2OS cells (data not shown). The inhibitory effects of BTG3 on AKT signaling, fibronectin and ZEB1 expression, the *β*-catenin/TCF reporter, and cell migration were also observed in PC3 cells overexpressing BTG3 (Supplementary Figure S3).

To further test whether BTG3-dependent inhibition of *β*-catenin was mediated through GSK3*β*, the GSK3-specific inhibitor CT99021 was used. As predicted, overexpression of BTG3 suppressed the activity of *β*-catenin/TCF, and such inhibition was abolished in the presence of CT99021 (Figure 3g), thus supporting the role of GSK3*β* in mediating the inhibitory effect of BTG3. Taken together, these results implicate BTG3 in the negative regulation of the AKT-GSK3*β*-*β*-catenin signaling axis.

### A BTG3-derived peptide recapitulates the activity of the full-length BTG3 protein in suppressing AKT

**Figure 4:**
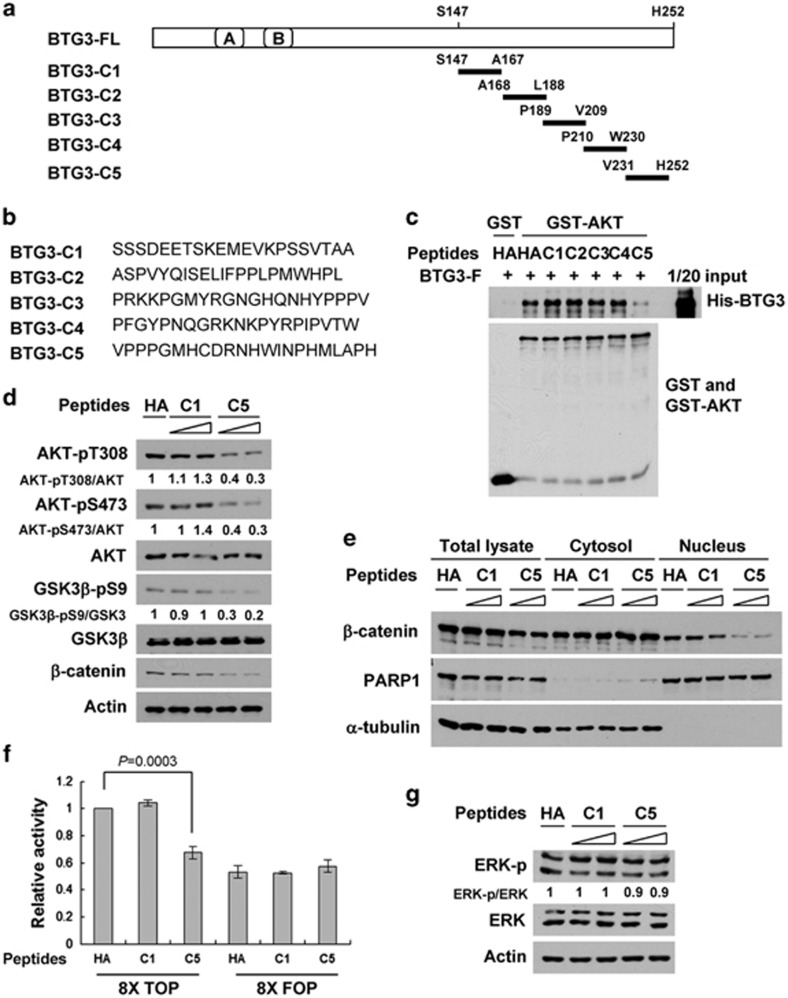
A peptide derived from the BTG3 C terminus recapitulates the activity of the full-length protein in antagonizing AKT. (**a**) Schematic representation of the BTG3 protein and its derived peptides. (**b**) Amino-acid sequences of the five C-terminal peptides C1 to C5. (**c**) The C5 peptide but not C1, C2, C3, or C4 competes with the His-tagged, full-length BTG3 protein for binding GST-AKT *in vitro*. (**d**) The C5 but not the C1 peptide suppresses AKT in cells. The 293 T cells were transfected with the indicated peptides, and the resulting lysates were analyzed by immunoblotting using the indicated antibodies. The unrelated HA peptide was used as a negative control for comparison. (**e**) Nuclear *β*-catenin was reduced in cells transfected with the C5 but not the C1 peptide. (**f**) Transcriptional activity of *β*-catenin/TCF was diminished in C5- but not in C1-transfected cells. (**g**) Neither the C5 nor the C1 peptide affected ERK phosphorylation in 293 T cells

To understand further the molecular basis of AKT interaction and suppression, five peptides (C1–C5) spanning the BTG3 C-terminal AKT interaction domain (amino acids 147–252) were synthesized (Figures 4a and b). These peptides were first tested for their ability to compete with the full-length BTG3 for binding AKT in GST pull-down assays. Among the five peptides tested, only C5 was able to compete and interfere with the interaction between BTG3 and AKT (Figure 4c). Consistently, transfected C5 but not C1 or the control unrelated influenza hemagglutinin (HA) peptide suppressed the phosphorylation of AKT and GSK3*β* in 293 T cells (Figure 4d), indicating that the C5 peptide might functionally mimic the full-length BTG3 protein. In support of this idea, the levels and the activity of nuclear *β*-catenin were reduced in the C5 but not in the C1 peptide-transfected cells (Figures 4e and f). As a control, neither the C5 nor the C1 peptide had any detectable effect on extracellular signal-regulated protein kinase (ERK) phosphorylation (Figure 4g), thus demonstrating the specificity of the regulation. These effects were reproduced with a C5-derived peptide spanning the C-terminal (C5-C) but not the N-terminal half (C5-N) of C5 (Supplementary Figure S4), thus revealing a minimal AKT-interacting motif in the region between amino acids 241 and 252.

### Identification of residues in the BTG3 C-terminal peptide critical for inhibition of AKT

**Figure 5:**
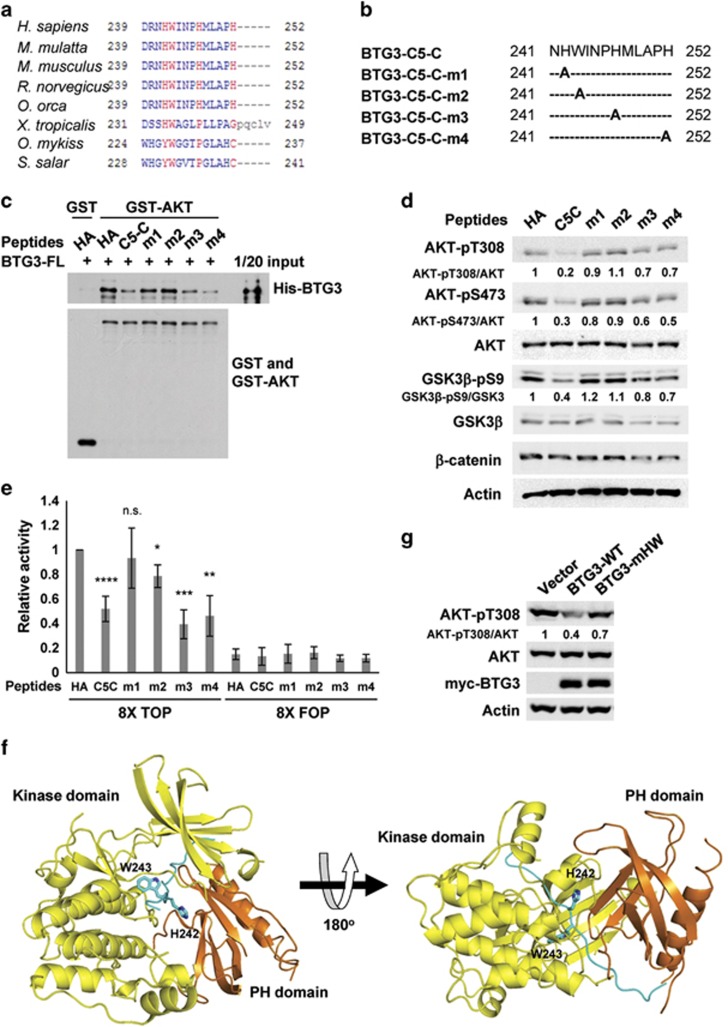
Identification of BTG3 C-terminal residues critical for AKT suppression. (**a**) Alignment of the C termini of BTG3 from different species. Residues with high conservation are shown in red. (**b**) Schematic representation showing the Ala substitution in the C5-C mutants. (**c** and **d**) An Ala substitution at position H242 or W243 attenuates the inhibitory activity of the C5-C peptide in an *in vitro* interaction assay (**c**) and on AKT-GSK3*β* signaling in 293 T cells (**d**). (**e**) Inhibition of the *β*-catenin/TCF reporter activity by C5-C was abrogated by Ala substitution at H242 and to a lesser extent at W243. *P<0.05, **P<0.01, ***P<0.001, and ****P<0.0001, respectively, by Student's *t*-test. (**f**) Docking model of the BTG3 C-terminal peptide (residues M236–H252) in the interdomain region of AKT1. The peptide (blue) sits in the interdomain pocket between the PH domain (orange) and the kinase domain (yellow), locking the two domains in a ‘PH-in' conformation. (**g**) Simultaneous mutation of H242 and W243 to Ala in the context of the full-length protein attenuates the inhibitory activity of BTG3 on AKT in 293 T cells

Based on the study by Wu *et al.*^30^ in which a small-molecule AKT inhibitor with aromatic ring structures was shown to lock the PH and the kinase domain of AKT in an inactive conformation, we speculated that a residue or residues with similar structure(s) in C5-C might act similarly. By aligning the regional amino-acid sequences of BTG3 from different species, we found that H242 and W243 were conserved in mammals and amphibians. The other two ring-type residues, H247 and H252, although conserved among mammals, were not present in frog BTG3 (Figure 5a). We therefore conducted alanine substitution at four residues: H242, W243, H247, and H252 (Figure 5b). Interestingly, mutation at H242 and W243 abolished the inhibitory activity of C5-C, whereas mutation at H247 and H252 had no apparent effect on BTG3-AKT interaction (Figure 5c), AKT signaling (Figure 5d), and the *β*-catenin/TCF reporter activity (Figure 5e). An *in silico* model fitting was then attempted. The deduced model indicated that, similar to the AKT inhibitor, residues H242 and W243, when docked to the AKT1 PH kinase interdomain region (Protein Data Bank, PDB code 3O96) in the context of the C5 peptide, were able to make close contacts with functional groups stemming from the PH and the kinase domains (Figure 5f), suggesting a similar mode of AKT1 inactivation.

To verify the importance of residues H242 and W243 in the context of the full-length protein, we then generated a mutant BTG3 with these two residues mutated to A. Similar to the mutant peptide, the BTG3 AA mutant (BTG3-mHW) was less efficient in suppressing AKT phosphorylation when compared with the wild-type protein (Figure 5g). Our study thus identified at least two residues in the C terminus of BTG3 that mediate the inhibition of AKT.

### BTG3 suppresses cell growth in three-dimensional culture

**Figure 6:**
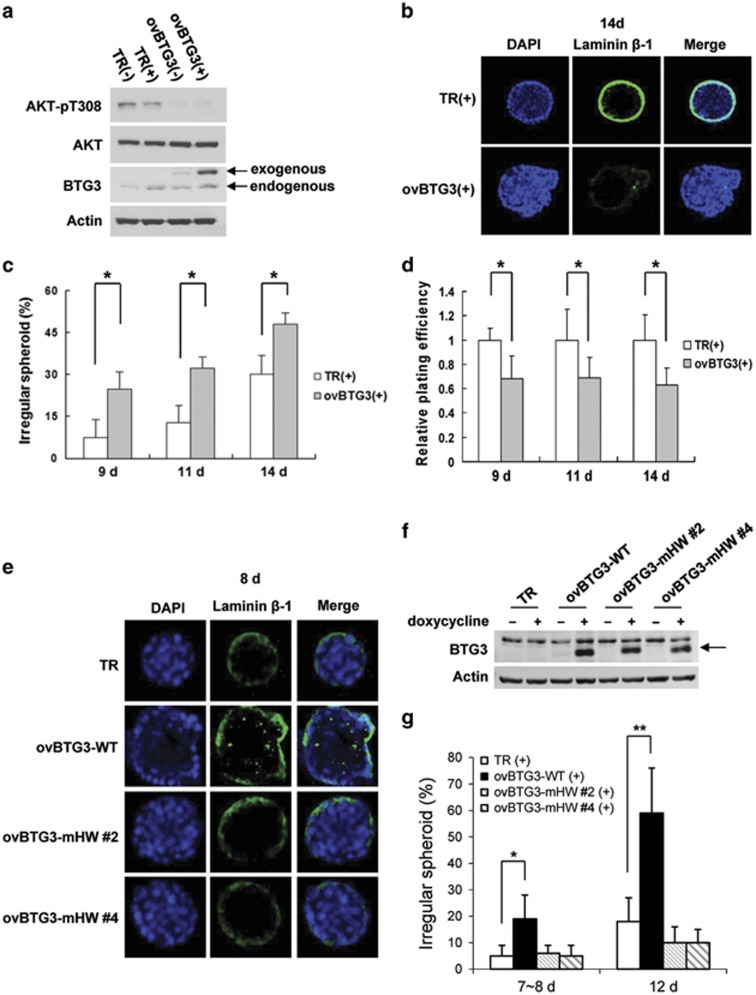
BTG3 suppresses growth of PC3 prostate cancer cells in 3D culture. (**a**) Characterization of PC3 Tet-On cells that express myc-tagged BTG3 upon the addition of doxycycline (+). TR, control cells that express only the tetracycline regulator; ovBTG3, Tet-On BTG3-overexpressing cells (clone no. 6). AKT T308 phosphorylation (pT308) was downregulated in ovBTG3 cells. (**b**) Immunofluorescence microscopy of spheroids grown in 3D culture. Shown are representative images of spheroids undergoing polarized differentiation, forming a lumen (upper panel); and spheroids with disrupted polarization (lower panel). (**c** and **d**) BTG3 overexpression disrupted the polarized growth of PC3 cells (**c**), and reduced the plating efficiency in 3D culture (**d**). The mean±S.D. of three independent experiments is shown. (**e–g**) The BTG3-mHW mutant failed to disrupt the polarized growth in 3D culture. Representative microscopic images are shown in (**e**), and expression of the wild-type (WT) and mHW BTG3 proteins in Tet-On PC3 cells, as examined by western blotting, is shown in (**f**). Quantitative results from four independent experiments are shown in (**g**). *P<0.05 and **P<0.01, respectively, by Student's *t*-test

Three-dimensional (3D) matrix culture has been shown to mimic more closely the stromal microenvironment *in vivo* than 2D culture, and to allow phenotypic discrimination between malignant and nonmalignant cells.^31, 32^ As a step toward understanding the role of BTG3 in tumor progression, we first determined its impact on cells grown in a 3D matrix. For this assay, we chose to use PC3 cells that lack PTEN and have high AKT activity. Thus, we established PC3 Tet-On cell lines (ovBTG3) that inducibly express an exogenous myc-tagged BTG3 upon the addition of doxycycline (Figure 6a). Consistent with our observations in other cells, AKT phosphorylation in these cells was markedly reduced (Figure 6a). A significant reduction in AKT phosphorylation in ovBTG3 cells was also observed in the absence of doxycycline, likely because of leaky expression. The growth rates of the TR (control cells that express only the tetracycline regulator) and ovBTG3 cells in a 3D matrix (Matrigel) were compared. As previously described for PC3 cells,^33^ PC3-TR cells grew in spheroids on Matrigel, underwent polarized differentiation, and formed acinar structures. By contrast, the PC3-ovBTG3 cells formed mostly irregular spheroids and incompletely developed lumens (Figures 6b and c), suggesting a disrupted polarization. Furthermore, the plating efficiency was significantly reduced with the ovBTG3 cells compared with the control PC3-TR cells (Figure 6d). However, despite these differences, both cell lines formed invasive stellates upon prolonged incubation (more than 2 weeks, data not shown), suggesting that, at least in PC3 cells, although BTG3 overexpression alone is capable of suppressing AKT, it is insufficient in inhibiting invasive growth in 3D culture. Similar results were obtained with another PC3-ovBTG3 clone (Supplementary Figure S5), thus excluding possible clonal effects. Collectively, these results suggest that BTG3 not only dampens cell colonization but also appears to hamper the polarization of PC3 cells in the 3D matrix.

To link the above observation with the regulation of AKT, we then compared in 3D culture the activity of BTG3 WT and the BTG3-mHW mutant impaired in AKT inhibition. Our data showed that unlike the PC3-ovBTG3 WT, two of the PC3-ovBTG3-mHW (nos. 2 and 4) clones grew in regular spheroids as the control PC3-TR cells (Figures 6e and g), despite comparable levels of BTG3 were expressed (Figure 6f). This result thus demonstrated a functional relevance of the residues H242 and W243 in growth in addition to their involvement in the regulation of AKT phosphorylation and activation.

### BTG3 suppresses tumor growth in a xenograft mouse model

**Figure 7:**
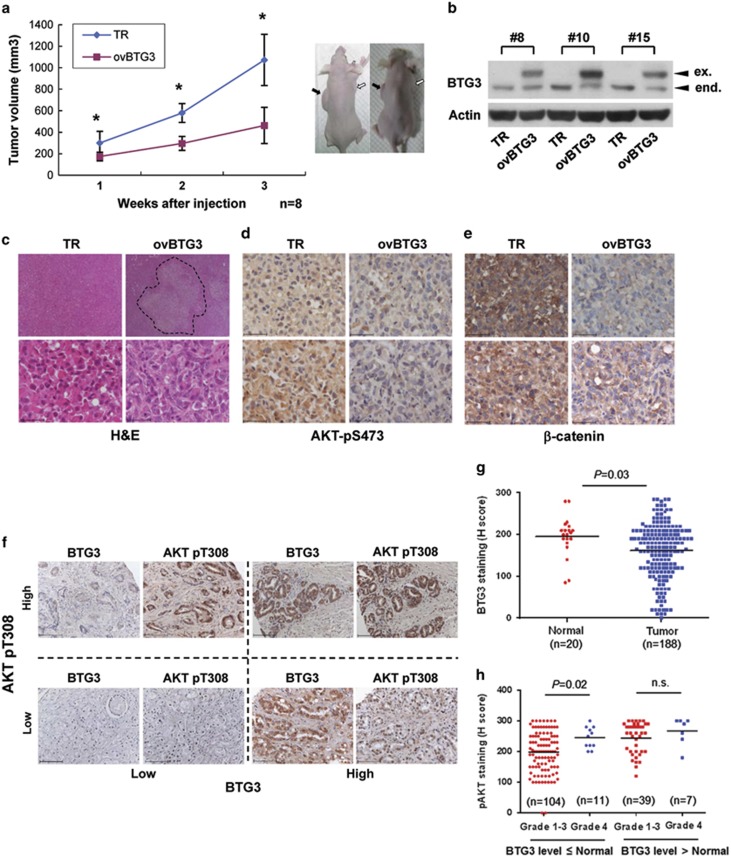
BTG3 overexpression suppresses tumor growth. (**a**) BTG3 impedes the growth of xenograft tumors. PC3-TR- or BTG3-overexpressing (ovBTG3) cells (clone no.19) were mixed with Matrigel and injected subcutaneously into athymic (nude) mice. The xenograft tumors were removed 4 weeks after injection. Left: Weekly comparison of the volume of control TR and ovBTG3 tumors. *P<0.01. Right: Mice with the xenograft tumors. Arrows on the left denote PC3-TR tumors; on the right, PC3-ovBTG3 tumors. (**b**) BTG3 expression in excised tumors. Tumor tissue extracts were analyzed by western blotting with the antibody against BTG3. Actin was used as a loading control. Numbers 8, 10 and 15 denote mouse ID. (**c**) Representative hematoxylin and eosin (H&E) staining of the tissues from the TR (left) and the ovBTG3 (right) xenograft tumors. The necrotic area is outlined. Original magnification × 12.5 (top) and × 400 (bottom). (**d** and **e**) Immunohistochemical (IHC) staining of tumor sections with anti-phospho-AKT Ser473 (**d**) and anti-*β*-catenin (**e**). Representative staining from two different mice, upper and lower panels, respectively, is shown. Scale bar, 50 *μ*m. (**f**) Expression of BTG3 and phospho-AKT in human prostate cancer specimens. Tissue sections of paraffin-embedded prostate cancer specimens were stained with anti-BTG3 or anti-AKT pT308 antibodies and counterstained with hematoxylin. Multiple paired BTG3 and AKT pT308 stainings are shown. Scale bar, 100 *μ*m. (**g**) BTG3 is downregulated in prostate cancer. The intensity of staining in (**f**) was quantified using *H*-score and compared with respect to BTG3 between normal and tumor tissues. (**h**) Phospho-AKT (pT308) is increased as disease progresses in BTG3-downregulated prostate cancer. The intensity of AKT pT308 staining was compared in tumors with (≦Normal ) or without (>Normal) BTG downregulation, separated using mean BTG3 staining in normal tissues. Data were analyzed by Student's *t*-test. NS, nonsignificant

To determine the role of BTG3 in tumor suppression in a more physiological setting, we performed xenograft studies by implanting the PC3-TR (control) or PC3-ovBTG3 cells subcutaneously into immunosuppressed nude mice. The tumors produced were compared for volume and histology. As shown in Figure 7a, tumors produced by PC3-ovBTG3 cells were smaller than those from the PC3-TR control cells. Analysis of lysates prepared from the tumors showed that they retained the ectopically expressed BTG3 (Figure 7b). Histologically, some of the PC3-ovBTG3 tumors displayed a large area of necrosis, mostly at the core and some at the periphery of the tumor, which might account for their smaller size (Figure 7c, upper panel). Immunohistochemical staining also confirmed reduced phospho-AKT (pS473) (Figure 7d) and *β*-catenin levels (Figure 7e) in PC3-ovBTG3 tumors, thus providing further support to our cell-based studies. These observations were reproduced with another PC3-ovBTG3 clone in an independent experiment (Supplementary Figure S6), thus excluding possible clonal effects.

### Increased AKT phosphorylation in human prostate cancer specimens with reduced BTG3 expression

To determine the clinical relevance of the BTG3-AKT axis, we performed IHC staining on paraffin-embedded sections of clinical prostate cancer specimens to compare the levels of BTG3 and phospho-AKT (pT308) (Figure 7f). Of the 188 tumor tissue sections (from 94 cases) and 20 normal/adjacent normal tissue sections (from 20 cases) examined, significant reduction in BTG3 staining was found in tumor tissues compared with normal tissues (*P*=0.03) (Figure 7g). This is in agreement with our previous observation and with observations from others that BTG3 is downregulated in prostate cancers.^13, 14^ Within the BTG3-downregulated tumors (≦Normal; Figure 7h), it was observed that phospho-AKT (pT308) staining was increased as the disease progresses from differentiated or poorly differentiated (grades 1–3) to undifferentiated (grade 4) stage (*P*=0.02; Figure 7h). However, this trend was not as apparent in tumors expressing higher level of BTG3 (>Normal; Figure 7h), suggesting the existence of other AKT regulator in these tumors. These results highlight an inverse relationship between the levels of BTG3 and AKT activity with respect to disease progression, further supporting a likely involvement of BTG3-AKT regulation in prostate cancer.

## Discussion

Deregulation of the PI3K-AKT pathway is known to have a prominent role in various cancers, especially in the prostate.^34^ Here we demonstrated that BTG3 guards against the AKT-GSK3*β*-*β*-catenin signaling axis by binding and preventing AKT from localizing to the plasma membrane.

### BTG3 as a suppressor of tumorigenesis

The BTG protein family members are known to be antiproliferative; however, their role in tumor suppression has not been examined extensively. Loss of expression including loss of heterozygosity or promoter methylation of the *BTG* family genes have been reported in different types of cancers.^11, 12, 13, 35, 36, 37^ Knockout mouse models for BTG3 and Tob were established,^27, 38^ which either succumbed to lung tumors at an old age in the case of *Btg3* knockouts^27^ or spontaneously developed a variety of tumors in the case of *Tob* knockouts.^38^ Although increased cyclin D1 expression was implicated in tumor formation in the *Tob*-null mice, it was not clear why *Btg3* deficiency caused lung tumors in mice. Data presented in this report together with our recent study^14^ provide evidence that BTG3 acts to regulate at least two proproliferation pathways frequently deregulated in cancers: the MAPK (mitogen-activated protein kinase) and the AKT pathways. Thus, overexpression of BTG3 inhibited AKT signaling, and downregulation of BTG3 increased the levels of phospho-ERK and phospho-AKT. In normal cells, this initial response was inactivated soon after the engagement of the senescence program, whereas in genetically predisposed cells malignant transformation might be triggered, and we also showed that increased AKT signaling led to the inhibition of GSK3*β* and activation of *β*-catenin.

Although our PC3 xenograft study showed that BTG3 could be a tumor suppressor, a reciprocal knockdown in U2OS cells failed to promote xenograft tumors (data not shown). One reason that could account for this outcome might reside in the U2OS cells, which by themselves could not form tumors in mice (data not shown). Although BTG3 downregulation was shown to promote expression of EMT markers and cell migration in these cells, the effects appeared insufficient to drive tumor formation *in vivo*, at least for U2OS cells. One could interpret these negative results as meaning that the loss of BTG3 alone is not sufficient to drive tumorigenesis. This interpretation is supported by our recent finding that the loss of BTG3 could trigger senescence in normal cells^14^ and also by the findings of others that mice with transgenic AKT expression developed prostate intraepithelial neoplasia but not cancers.^39^ Thus, one could predict that additional events are required to trigger cancerous progression. To address its tumor suppressor role, a *Btg3* conditional allele that allows inducible ablation of *Btg3* in different cancer models may provide more physiologically relevant answers.

### BTG3 and PTEN in the defense against tumorigenesis

The data presented here and our recently published study regarding the role of BTG3 in genomic stability^10^ underscore the surprising functional similarities between BTG3 and PTEN. Both are known to be transactivated by p53 upon genotoxic stress,^4, 40^ and inactivation of either one impairs the checkpoint response.^4, 41^ Furthermore, both negatively regulate PI3K/AKT signaling, although different mechanisms are used in each case. This mechanistic difference may explain, at least in part, the differential impact on prostate cancer when they are lost. PTEN deficiency occurs in 60–80% of prostate cancers^42^ with a clear causal relationship. The impact of reduced BTG3 expression appears to be less pronounced. In addition, unlike *Pten*-null mice that died *in utero*,^43^ the *Btg3*-knockout mice developed normally and were not particularly tumor-prone until 21 months, at which time lung tumors were detected.^27^ The possibility that the *Btg3* deficiency was complemented by the other members of the gene family such as *Btg2* has yet to be excluded.

Another striking similarity between BTG3 and PTEN is their impact on CHK1. Loss of PTEN impairs CHK1 activity via AKT-dependent phosphorylation and its cytoplasmic sequestration.^41^ On the other hand, BTG3 safeguards CHK1 activation by promoting its K63-linked ubiquitination and chromatin association.^10^ Thus, through different pathways, both proteins appear to regulate the activity of CHK1. Additionally, as demonstrated here, BTG3 could indirectly promote CHK1 activity by inhibiting AKT activation.

### Development of a potential AKT inhibitor

Various inhibitors targeting the PI3K pathway have been developed; many of them are ATP (adenosine triphosphate)-competitive.^44^ The BTG3-derived, AKT inhibitory small peptide we identified here represents a distinct category of AKT inhibitors. As with some of the existing AKT inhibitors, the BTG3 peptide acts allosterically, most likely by keeping the kinase in a ‘PH-in'conformation^45^ as suggested by our data here. However, unlike those small-molecule AKT inhibitors, the small BTG3 peptide is based on a cellular protein and thus might have the advantage of lower cytotoxicity and higher specificity. By combining with a proper delivery system, a modified BTG3 peptide or its derivatives with improved stability could be developed. In light of the important role of AKT in tumorigenesis, angiogenesis, and metastasis, the BTG3 peptide or its derivatives may serve as promising leads for cancer drug development.

## Materials and Methods

### Cell lines

To establish inducible osteosarcoma U2OS and prostate cancer PC3 Tet-On stable cell lines for BTG3 knockdown or overexpression, U2OS-TR cells (tetracycline regulator-expressing)^46^ or PC3-TR cells were stably transfected with pBabe-H1-shBTG3^4^ or pcDNA4-TO-BTG3, respectively. The U2OS BTG-knockdown stable cell line was maintained regularly in complete DMEM containing 2.5 *μ*g/ml blasticidin (InvivoGen, San Diego, CA, USA) and 0.8 *μ*g/ml puromycin (Sigma-Aldrich, St. Louis, MO, USA), and the PC3 BTG3-overexpressed line in F-12 K plus 2.5 *μ*g/ml blasticidin and 100 *μ*g/ml Zeocin (InvivoGen). Induction was performed using 1 *μ*g/ml doxycycline (Sigma-Aldrich).

The human embryonic kidney 293T cells and the prostate cancer cell line DU145 were maintained, respectively, in DMEM and RPMI containing 10% fetal bovine serum (FBS), 100 U/ml penicillin, and 100 *μ*g/ml streptomycin (all from Gibco, Life Technologies, Grand Island, NY, USA).

### Plasmids and constructs

To generate GST-tagged N- and C-terminal BTG3 truncation constructs, the corresponding regions of BTG3 were amplified by PCR and cloned into the *Bam*HI and *Xho*I sites of the pGEX4T-1 vector (Amersham Biosciences, Piscataway, NJ, USA). The plasmids expressing GST-tagged AKT full-length and truncation mutants were generated by PCR amplification of the corresponding coding regions from pcDNA3-myr-HA-AKT (kindly provided by Jeffrey J-Y Yen, Institute of Biomedical Sciences, Academia Sinica, Taipei, Taiwan) and cloned between the *Eco*RI and *Xho*I sites of pGEX4T-1. For mammalian expression of HA-AKT, the full-length AKT was amplified by PCR from pcDNA3-myr-HA-AKT and cloned between the *Eco*RI and *Xho*I sites of the pcDNA3-HA vector. The myristoylation (myr) sequence was removed to allow dynamic regulation of AKT in cells. All sequences amplified by PCR were verified by DNA sequencing.

### GST pull-down assay

The assay was performed essentially as described previously.^46^ For peptide competition, 20 × molar excess of the peptide was included in the reaction to compete with the full-length BTG3 protein.

### Plasmid and siRNA transfection

Plasmids were transfected using Lipofectamine 2000 (Invitrogen Life Technologies, Carlsbad, CA, USA) except in 293 T cells, for which the calcium phosphate precipitation method was used. All siRNAs were synthesized by Sigma-Aldrich. Transfection of siRNA was performed using Oligofectamine (Invitrogen Life Technologies). The sequences targeted by the BTG3 siRNAs were 5′-GGCTAGTTCGAAAACATGA-3′ (BTG3-1) and 5′-TTGAGAGGTTTGCTGAGAA-3′ (BTG3-2).

### Peptide transfection

All peptides were commercially synthesized at over 70% purity (Kelowna International Scientific Inc., New Taipei, Taiwan). Cells were plated 1 day before transfection at a density that would reach ~50% confluence at the time of transfection. Peptides (0.5–1 μg/35 mm dish) were transfected using the TransPass P Transfection Reagent (New England BioLabs, Beverly, MA, USA) as directed.

### Cell lysis and immunoblotting

Cell lysates were prepared as described.^47^ Immunoblotting was carried out using the following antibodies against: actin (A2066), *α*-tubulin (T6074), and fibronectin (F6140) from Sigma-Aldrich; GST (sc-138), His (sc-803), and Myc (sc-40) from Santa Cruz Biotechnology (Santa Cruz, CA, USA); HA (Covance, Berkeley, CA, USA); *β*-catenin (no. 610154) and protein kinase B*α*/AKT1 (no. 610860) from BD Biosciences (San Jose, CA, USA); phospho-AKT (Thr308) (no. 4056), phospho-AKT (Ser473) XP (no. 4060), phospho-GSK-3*β* (Ser 9) (no. 9323), GSK-3*β* (no. 9315), vimentin (no. 3932), N-cadherin (no. 4061), Slug (no. 9585), and ZEB1 (no. 3396) from Cell Signaling Technologies (Danvers, MA, USA); and ERK1 and 2 (pTpY^185/187^) (no. 44680 G) and ERK1 and 2 (no. 44-654 G) from Invitrogen Life Technologies. The rabbit anti-BTG3 antibody has been described previously.^4^

### Cell fractionation

Cytosolic and nuclear fractions from control and BTG3-depleted U2OS cells were prepared as described previously.^48^ Membrane fractions were prepared essentially as described.^49^

### Co-immunoprecipitation

Lysis of cells and immunoprecipitation were carried out as described previously^10^ using anti-BTG3 or anti-AKT (no. 610860; BD Biosciences).

### Luciferase reporter assay

U2OS cells were first transfected with control or BTG3 siRNA using Oligofectamine (Invitrogen Life Technologies). The following day, cells were transfected with the *β*-catenin/TCF reporter 8 × FOPFlash (mutant) or 8 × TOPFlash (wild-type) (Addgene, Cambridge, MA, USA). Assays were performed as described previously.^4^

### Transwell migration assay

Cell migration was assayed in a 24-well Boyden chamber (Corning Costar, Corning, NY, USA). Equal numbers of Tet-On U2OS stable cells induced or not induced to express BTG3 shRNA were plated in serum-free medium in the upper compartment of the Boyden chamber. The lower compartment contained 700 *μ*l of 10% FBS in the medium, which was used as a chemoattractant. Cells that migrated through the insert after 16–18 h were fixed with 100% methanol and stained with Wright–Giemsa stain. Cells on the lower surface of each insert, in a total of five random objective fields, were counted using a light microscope at × 200 magnification. Student's *t*-test was applied to assess the significance of differences between mean counts.

### 3D culture

PC3 cells were trypsinized and resuspended in F12K medium (Invitrogen Life Technologies) containing 10% FBS, 1% Pen/Strep mix, and 2% Matrigel (BD Biosciences) to a final concentration of 5000 cells/ml. Two thousand cells were seeded into each well of an 8-well chamber slide (Millipore EZ; Millipore Corp., Bedford, MA, USA) precoated with 60 μl of Matrigel. The culture was refreshed with 400 μl medium every 4 days until the day of the assay. Irregular spheroids are judged based on the staining of 4',6-diamidino-2-phenylindole or laminin *β*1. Those that are not as rounded as the TR control and with short buds or sprouts are determined to be irregular. Plating efficiency was determined by the number of spheroids formed at the indicated time point divided by the number of cells plated on Matrigel. For protein analysis, spheroids in 3D culture were harvested and extracted as described.^31^

### Fluorescence microscopy

For membrane localization, 293 T cells were transfected with plasmids expressing either GFP or GFP-PH-AKT,^28^ together with either full-length BTG3 or the d4 mutant. Localization was analyzed by confocal microscopy and quantified using ZEN 2008 software (Carl Zeiss, Oberkochen, Germany).

For staining of the 3D culture, the spheroids were fixed in chamber slides with 4% paraformaldehyde for 30 min at room temperature before permeabilization. The anti-laminin *β*1 antibody (ab69633; Abcam, Cambridge, MA, USA) was used at 1 : 200 dilution. Images were captured using a Zeiss LSM510 confocal microscopy system (Carl Zeiss Microscopy GmbH, Jena, Germany).

### Generation of docking model

To derive the model, we first positioned H242 and W243 (blue sticks) of the peptide to the binding region of the imidazoquinoxaline and benzylpiperidine groups of the AKT inhibitor VIII (PDB code 3O96; Wu *et al.*^30^). This was followed by molecular dynamic simulation (100 ps) and energy minimization to relax the peptide and side-chain atoms of the protein at the binding site. The docking and simulation were carried out using Discovery Studio (Accelrys Inc., San Diego, CA, USA). Figure 5e was generated using PyMol (DeLano Scientific LLC, Palo Alto, CA, USA).

### Mouse xenograft studies

All animal studies were conducted according to the protocol approved by the Institutional Animal Care and Utilization Committee of Academia Sinica (AS IACUC, Protocol ID 11-12-271). Athymic (Bltw:NU-Foxn1nu) male mice (BioLASCO, Taipei, Taiwan) were maintained in pathogen-free conditions. Stable clones of TR- and BTG3-overexpressing PC3 cells (ovBTG3) (2 × 10^6^) were injected subcutaneously in 0.1 ml of Matrigel (BD Biosciences) into the backs of 4-week-old mice. Animals received doxycycline (200 *μ*g/ml) in the drinking water supplemented with 1% sucrose after the injection. The solution was refreshed two times a week and throughout the duration of the experiment. Tumor sizes were measured every week and the tumor volume was calculated using the formula *V*=0.5 × *a* × *b*^2^, where *a* is the length and *b* is the width (in mm).

### Immunohistochemistry

Human prostate cancer tissue microarrays (PR2085B; US Biomax Inc., Rockville, MD, USA) were stained as described previously,^14^ except that the slides were treated with 3% H_2_O_2_ before antigen retrieval. The antibodies used were anti-BTG3 (1 : 150; 4) and anti-AKT pT308 (1 : 250; sc-16646-R; Santa Cruz Biotechnology, Dallas, TX, USA). Images were taken using a Zeiss Imager A1 microscope (Carl Zeiss Microscopy GmbH). *H*-scores yielded from normal and tumor sections were then analyzed using Student's *t*-test.

For IHC staining of xenograft tumors, anti-phospho-AKT Ser473 (1 : 100; no. 3787; Cell Signaling Technologies) and anti-*β*-catenin antibodies (1 : 100; no. 610154; BD Biosciences) were used.

## Abbreviations

BTG: B-cell translocation gene
CHK1: checkpoint kinase 1
EMT: epithelial-to-mesenchymal transition
ERK: extracellular signal-regulated protein kinase
GFP: green fluorescent protein
GSK3β: glycogen synthase kinase 3*β*
Grp1: general receptor of phosphoinositides 1
HA: hemagglutinin
mTOR: mammalian target of the rapamycin
myr: myristoylation
PDK1: phosphoinositide-dependent kinase-1
PI3K: phosphoinositide 3-kinase
PTEN: phosphatase and tensin homolog
TCF: T-cell factor
ZEB1: zinc-finger E-box-binding homeobox 1
