# Supplementary material for: Candidate tumor suppressor B-cell translocation gene 3 impedes neoplastic progression by suppression of AKT
Source: Cell Death Dis. 2015 Jan 8;6(1):e1584–. doi: 10.1038/cddis.2014.550 (PMC4669748; doi:10.1038/cddis.2014.550)
Supplement: Supplementary Information [file cddis2014550x1.pdf]

Supplementary Information

Inventory of Supplementary Information

Supplementary Figures and Figure Legends

Figure S1 (supporting Figure 1)

Figure S2 (controls for Figure 2)

Figure S3 (supporting Figure 3)

Figure S4 (supporting Figure 5)

Figure S5 (supporting Figure 6)

Figure S6 (supporting Figure 7)

Supplementary Figures and Figure Legends

Figure S1 The BTG3 C-terminal region between amino acids 147 and 252 mediates direct interaction with AKT *in vitro*. (**a**) Schematic diagrams depicting various BTG3 constructs. (**b**) GST pull-down assays. Experiments were conducted with recombinant GST-BTG3 proteins, either full-length (FL), d4 (amino acids 147-252 deleted), or d6 (amino acids 108-146 deleted), and 293T lysates expressing HA-AKT. Proteins pulled down were analyzed by western blotting using the indicated antibodies.

Figure S2 BTG3 does not impact on the membrane recruitment of the Grp1 PH domain. **(a)** Membrane localization of the GFP-fused GrP1 PH domain (GFP-PH-Grp1) was not affected by either full-length BTG3 (FL) or the BTG3-d4 mutant. Confocal microscopy was performed on 293T cells transfected with indicated constructs. The fluorescence intensity in the membrane as a fraction of the total is shown in **(b)**. n.s., not significant. **(c)** Western blots showing relative expression levels of the two BTG3 proteins used in (a).

Figure S3 BTG3 suppresses AKT-GSK3β-β-catenin signaling in PC3 cells. **(a)** Overexpression of BTG3 downregulates phosphorylation of AKT and GSK3β. Cells were transfected with myc-BTG3. Lysates were analyzed by western blotting using the indicated antibodies. **(b)** Altered expression of EMT markers by overexpression of BTG3. Numbers are from normalization to Actin. **(c)** The β-catenin/TCF reporter activity was inhibited by BTG3 overexpression. **(d)** Cell migration was reduced upon BTG3 expression, as assessed by transwell assay.

Figure S4 The C-terminal half of the BTG3 C5 peptide is responsible for suppression of AKT–GSK3β–β-catenin signaling. **(a)** Schematic representation of full-length BTG3 and the two C-terminal peptides C5-N and C5-C. **(b)** Amino acid sequences of

C5-N and C5-C. **(c)** C5-C but not C5-N competes with the His-tagged, full-length BTG3 protein for binding GST-AKT *in vitro*. An unrelated peptide HA was used as a control. **(d)** C5-C but not C5-N inhibits AKT in cells. 293T cells were transfected with the indicated peptide, and the resulting lysates were analyzed by immunoblotting using indicated antibodies. **(e)** Nuclear β-catenin in 293T cells was more effectively downregulated with the C5-C peptide than with the C5-N peptide. **(f)** β-Catenin/TCF activity was diminished in C5-C- but not C5-N-transfected cells. **(g)** ERK phosphorylation in 293T cells was not affected by either C5-C or C5-N.

Figure S5 BTG3 suppresses growth of PC3 prostate cancer cells in 3D culture. **(a)** Characterization of PC3 Tet-On cells that express myc-tagged BTG3 upon the addition of doxycycline (+). TR, control cells that express only the tetracycline regulator. ovBTG3, Tet-On BTG3-overexpressing cells (clone #19). AKT T308 phosphorylation was downregulated in ovBTG3 cells. Leaky expression of BTG3 was observed before the addition of doxycycline, which may account for the decreased phospho-AKT T308. (**b, c**) BTG3 overexpression disrupted the polarized growth of PC3 cells (b), and caused reduced plating efficiency in 3D culture (c). The mean ± SD of three independent experiments is shown. *, $P < 0.05$ by Student's *t*-test.

Figure S6 BTG3 overexpression suppresses tumor growth. **(a)** BTG3 impedes the growth of xenograft tumors. PC3-TR or BTG3-overexpressing (ovBTG3) cells (clone #6) were injected s.c. into athymic (nude) mice as in Fig. 7, and tumors were removed 4 w after injection. Left: weekly comparison of the volume of control TR and ovBTG3 tumors. Right: mice with the xenograft tumors. Arrows on the left denote PC3 ovBT3 tumors; on the right, PC3 control TR tumors. *, $P<0.05$. **(b)** BTG3 expression in excised tumor. Tumor tissue extracts prepared from three different mice were analyzed by western blotting with the BTG3 antibody. Actin was used as loading control. #95, #97, and #99 denote mouse ID. **(c)** Representative hematoxylin

and eosin (H&E) staining of tissues from the TR (left) and the ovBTG3 (right) xenograft tumors. Original magnifications: 12.5 × (top) and 400 × (bottom). **(d, e)** IHC staining of tumor sections with anti-phospho-AKT Ser473 (d) and anti-β-catenin antibodies (e). Scale bar, 50 μm.

Cheng_FigS1

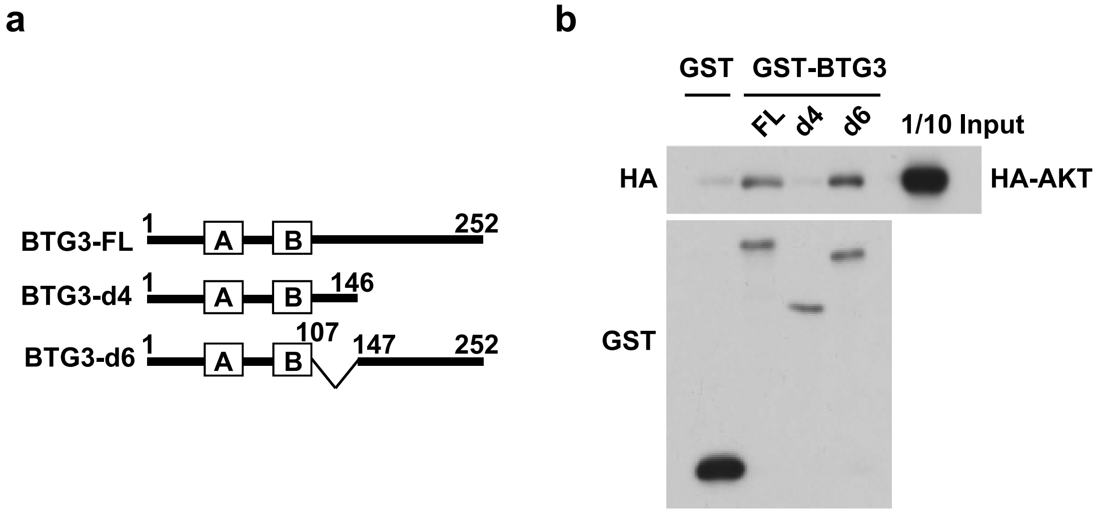
a
BTG3-FL
1
A
B
252
BTG3-d4
1
A
B
146
BTG3-d6
1
A
B
107
147
252
b
GST
GST-BTG3
FL
d4
d6
1/10 Input
HA
HA-AKT
GST

Cheng_FigS2

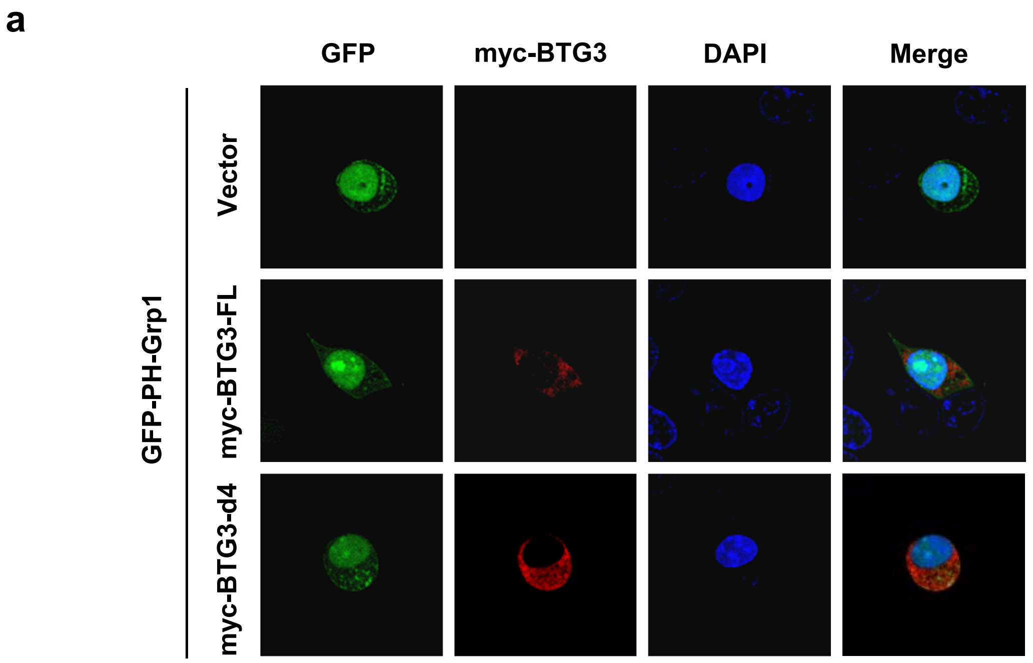

a
GFP
myc-BTG3
DAPI
Merge
GFP-PH-Grp1
Vector
myc-BTG3-FL
myc-BTG3-d4

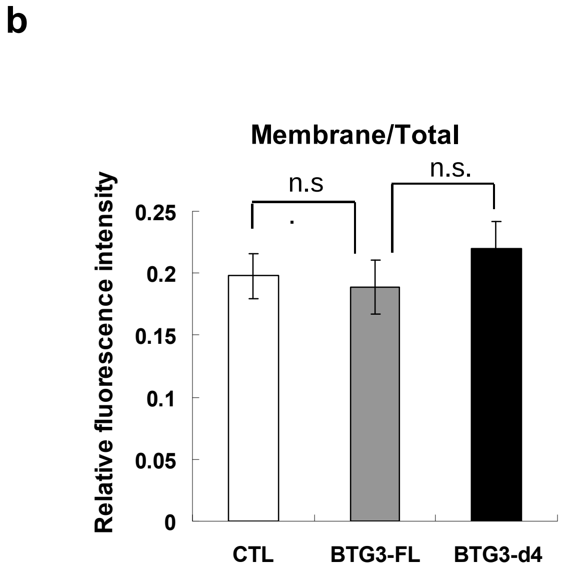

b
Membrane/Total
n.s
n.s.
Relative fluorescence intensity
0.25
0.2
0.15
0.1
0.05
0
CTL
BTG3-FL
BTG3-d4

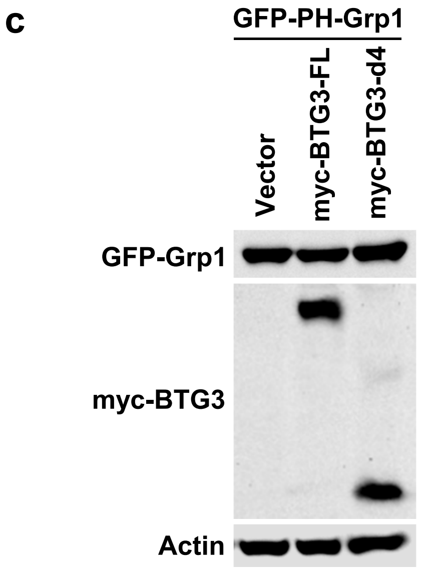

c
GFP-PH-Grp1
Vector
myc-BTG3-FL
myc-BTG3-d4
GFP-Grp1
myc-BTG3
Actin

Cheng_FigS3

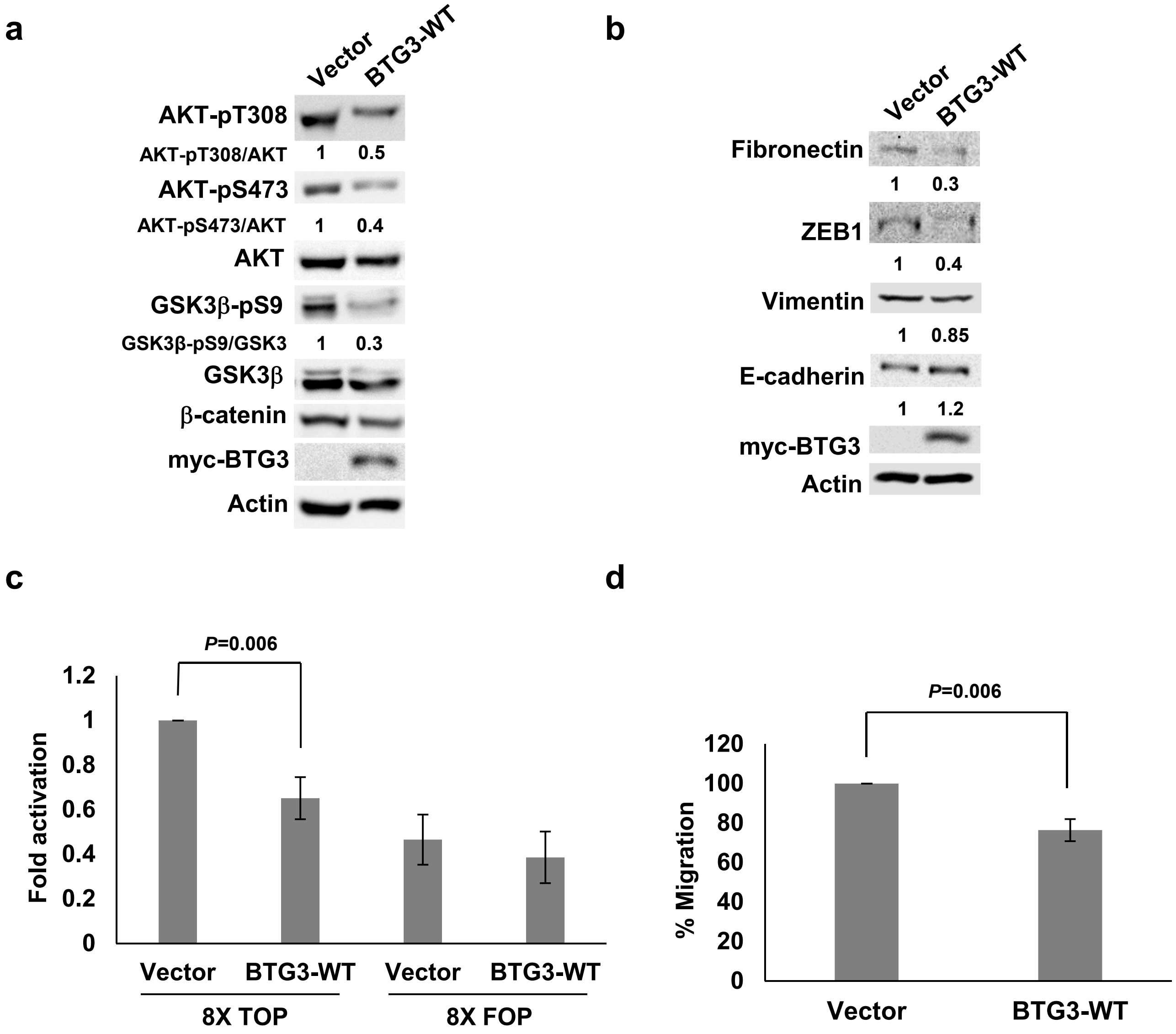
a
Vector
BTG3-WT
AKT-pT308
AKT-pT308/AKT 1 0.5
AKT-pS473
AKT-pS473/AKT 1 0.4
AKT
GSK3β-pS9
GSK3β-pS9/GSK3 1 0.3
GSK3β
β-catenin
myc-BTG3
Actin
b
Vector
BTG3-WT
Fibronectin
1 0.3
ZEB1
1 0.4
Vimentin
1 0.85
E-cadherin
1 1.2
myc-BTG3
Actin
c
P=0.006
Fold activation
1.2
1
0.8
0.6
0.4
0.2
0
Vector
BTG3-WT
Vector
BTG3-WT
8X TOP
8X FOP
d
P=0.006
% Migration
120
100
80
60
40
20
0
Vector
BTG3-WT

Cheng_FigS4

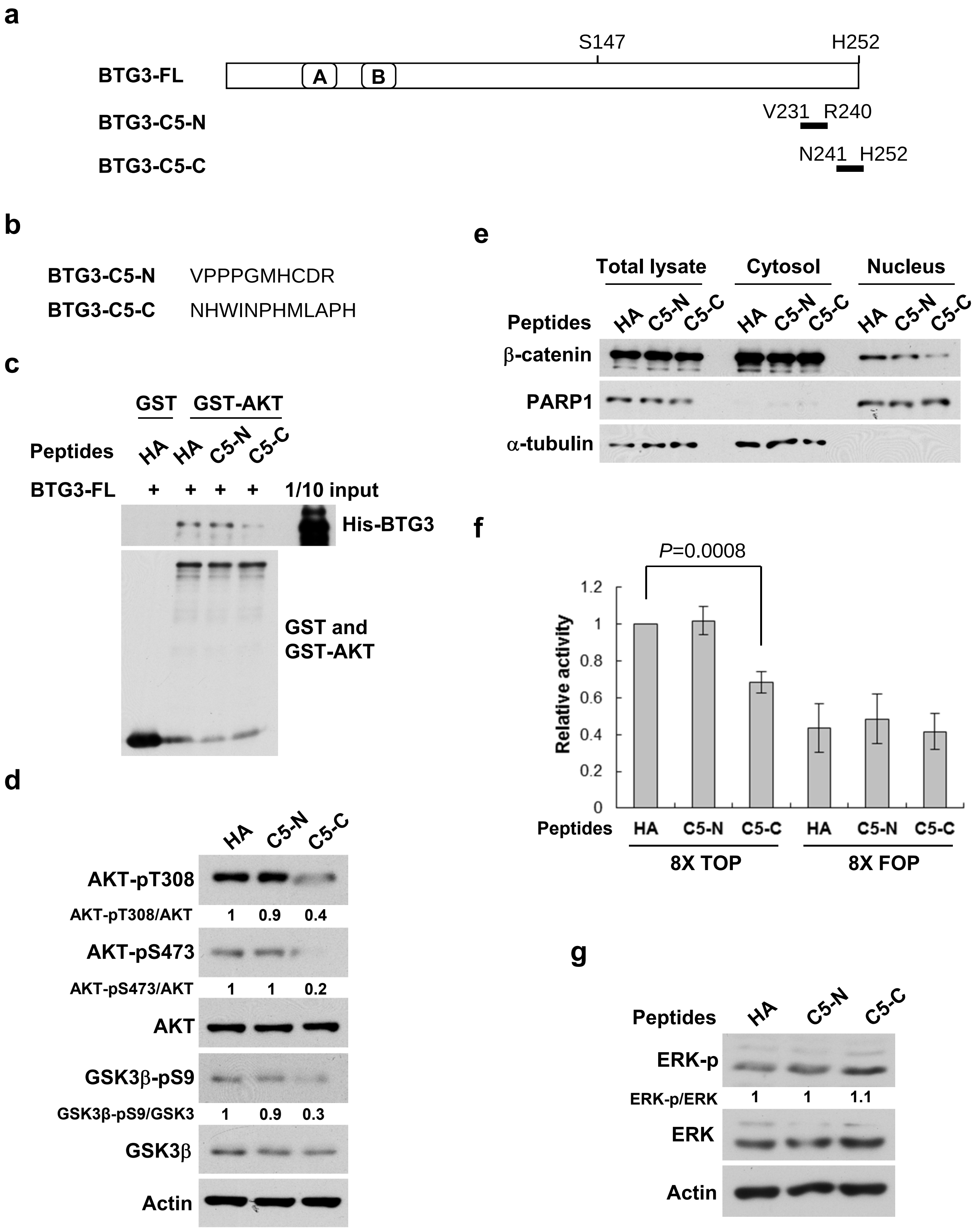
a
S147
H252
BTG3-FL
A
B
BTG3-C5-N
V231_R240
BTG3-C5-C
N241_H252
b
BTG3-C5-N VPPPGMHCDR
BTG3-C5-C NHWINPHMLAPH
c
GST
GST-AKT
Peptides HA HA C5-N C5-C
BTG3-FL + + + + 1/10 input
His-BTG3
GST and GST-AKT
d
HA C5-N C5-C
AKT-pT308
AKT-pT308/AKT 1 0.9 0.4
AKT-pS473
AKT-pS473/AKT 1 1 0.2
AKT
GSK3β-pS9
GSK3β-pS9/GSK3 1 0.9 0.3
GSK3β
Actin
e
Total lysate
Cytosol
Nucleus
Peptides HA C5-N C5-C HA C5-N C5-C HA C5-N C5-C
β-catenin
PARP1
α-tubulin
f
P=0.0008
1.2
1
0.8
0.6
0.4
0.2
0
Relative activity
Peptides HA C5-N C5-C HA C5-N C5-C
8X TOP
8X FOP
g
Peptides HA C5-N C5-C
ERK-p
ERK-p/ERK 1 1 1.1
ERK
Actin

Cheng_FigS5

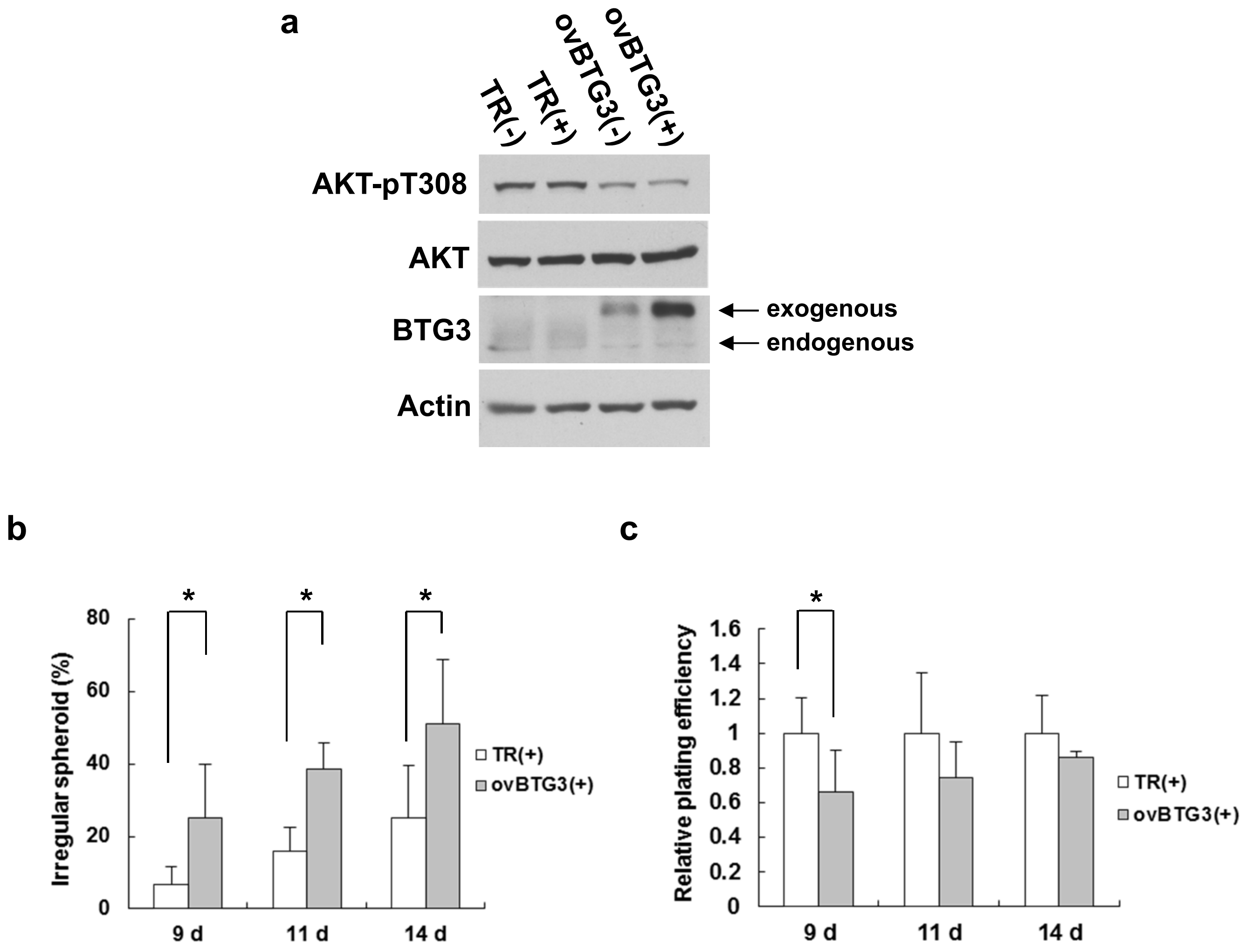
a
TR(-)
TR(+)
ovBTG3(-)
ovBTG3(+)
AKT-pT308
AKT
BTG3
exogenous
endogenous
Actin
b
Irregular spheroid (%)
0
20
40
60
80
*
*
*
TR(+)
ovBTG3(+)
9 d
11 d
14 d
c
Relative plating efficiency
0
0.2
0.4
0.6
0.8
1
1.2
1.4
1.6
*
TR(+)
ovBTG3(+)
9 d
11 d
14 d

Cheng_FigS6

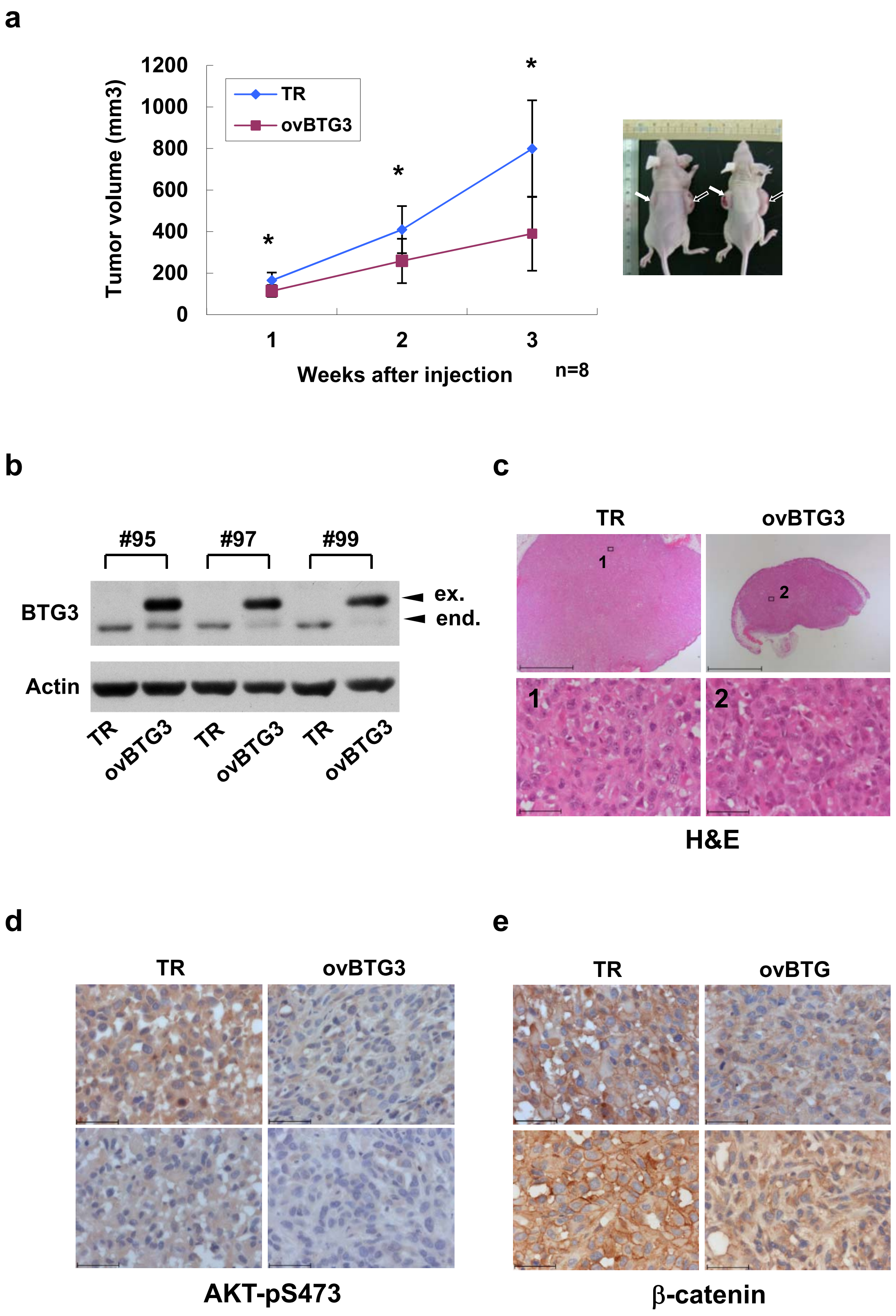
a
Tumor volume (mm3)
1200
1000
800
600
400
200
0
TR
ovBTG3
*
1
2
3
Weeks after injection
n=8
b
#95
#97
#99
BTG3
Actin
ex.
end.
TR
ovBTG3
c
TR
ovBTG3
1
2
H&E
d
TR
ovBTG3
AKT-pS473
e
TR
ovBTG
β-catenin
